# 

**Published:** 1885-07

**Authors:** 


					﻿Entered at the Post Office, at Austin, Texas, as Second Class Mail Matter
Vol. 1]
JULY, 1885.
NO. 1.
DANIEL'S
MEDICAL
JOURNAL
F. E. DANIEL., M. D., Editor and Publisher, AUSTIN, TEXAS.
EUGENE VON BOECKMANN, PRLNTER, AUSTIN, TEXAS.
				

